# Supplementary material for: Specific inhibition of splicing factor activity by decoy RNA oligonucleotides
Source: Nat Commun. 2019 Apr 8;10:1590. doi: 10.1038/s41467-019-09523-0 (PMC6453957; doi:10.1038/s41467-019-09523-0)
Supplement: Supplementary file 1 — Supplementary Information [file 41467_2019_9523_MOESM1_ESM.pdf]

Supplementary information:

Specific inhibition of splicing factor activity by decoy RNA oligonucleotides

Denichenko et al.

Supplementary Figures 1-12

Supplementary Table 1

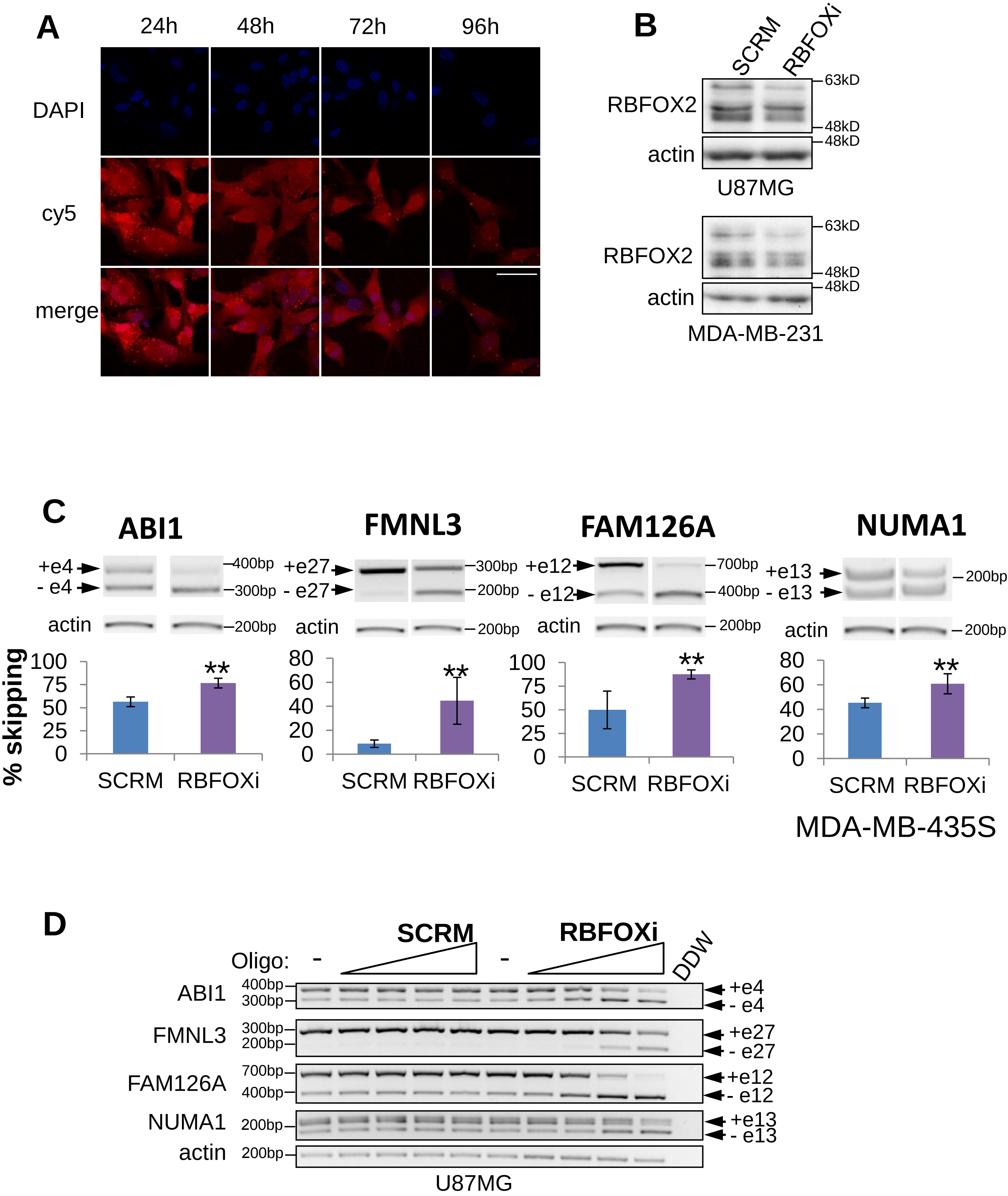

A
24h
48h
72h
96h
DAPI
cy5
merge
B
SCRM
RBFOXi
RBFOX2
63kD
48kD
actin
48kD
U87MG
RBFOX2
63kD
48kD
actin
48kD
MDA-MB-231
C
ABI1
FMNL3
FAM126A
NUMA1
+e4
- e4
400bp
300bp
+e27
- e27
300bp
200bp
+e12
- e12
700bp
400bp
+e13
- e13
200bp
actin
200bp
% skipping
**
SCRM
RBFOXi
MDA-MB-435S
D
SCRM
RBFOXi
Oligo:
-
DDW
ABI1
FMNL3
FAM126A
NUMA1
actin
U87MG

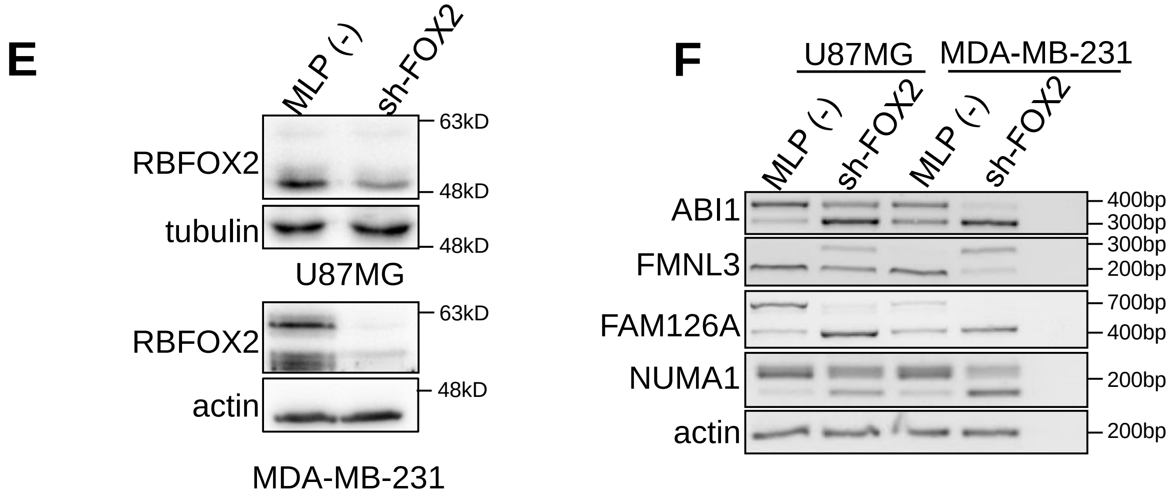

Supplementary Figure 1. Localization and splicing effects of RBFOX1/2 decoy oligonucleotides. (**A**) Fluorescent confocal microscopy of U87MG cells 24-96 hours after transfection with Cy5 modified RBFOXi. Blue- DAPI, red- Cy5. White line indicates 50µm. (**B**) Western blot of RBFOX2 in U87MG and MDA-MB-231 cells transfected with either 2.5µM RBFOXi or SCRM. (**C**) RT-PCR and quantification of known RBFOX1/2 splicing targets in MDA-MB-435S cells transfected with 2.5µM of either SCRM or RBFOXi (n=5). Gel image of representative experiment is shown above each column. The same actin control is shown for all panels, as the gels are from the same experiment. ** p-value < 0.004. (**D**) RT-PCR of known RBFOX1/2 targets in U87MG cells transfected with increasing amounts (0.5µM, 1µM, 2.5µM and 5µM) of RBOXi or SCRM. (**E**) Western blot of RBFOX2 in U87MG and MDA-MB-231 cells knocked down for RBFOX2 with shFOX2. (**F**) RT-PCR of known RBFOX1/2 splicing targets in RBFOX2 knocked down U87MG and MDA-MB-231 cells.

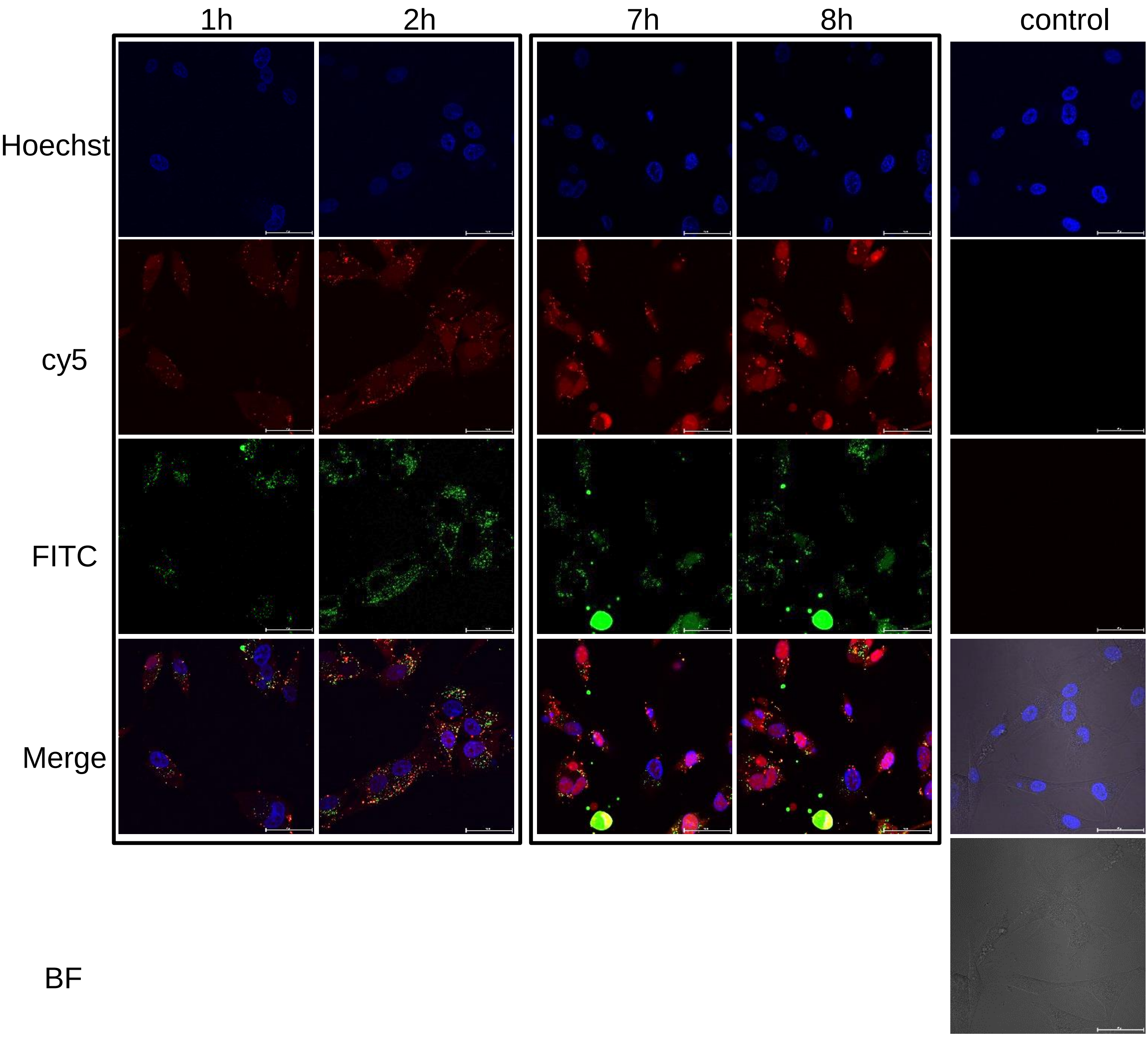

Supplementary Figure 2. Imaging of U87MG cells transfected with cy5 RBFOXi and endosome labeling with dextran.

Representative images of U87MG cells transfected with 2.5µM cy5 RBFOXi and 0.3mg/ml 488 alexa fluor marked dextran 1h, 2h (experiment 1) and 7h and 8h (experiment 2) after transfection and dextran treatment. Control (experiment 1) shows U87MG cells labeled with only Hoechst. Images were taken by Nikon SMZ18 stereomicroscope using NIS–Elements Br software. Blue- Hoechst (blue), cy5 RBFOXi (red), dextran (green). White line indicates 50µm.

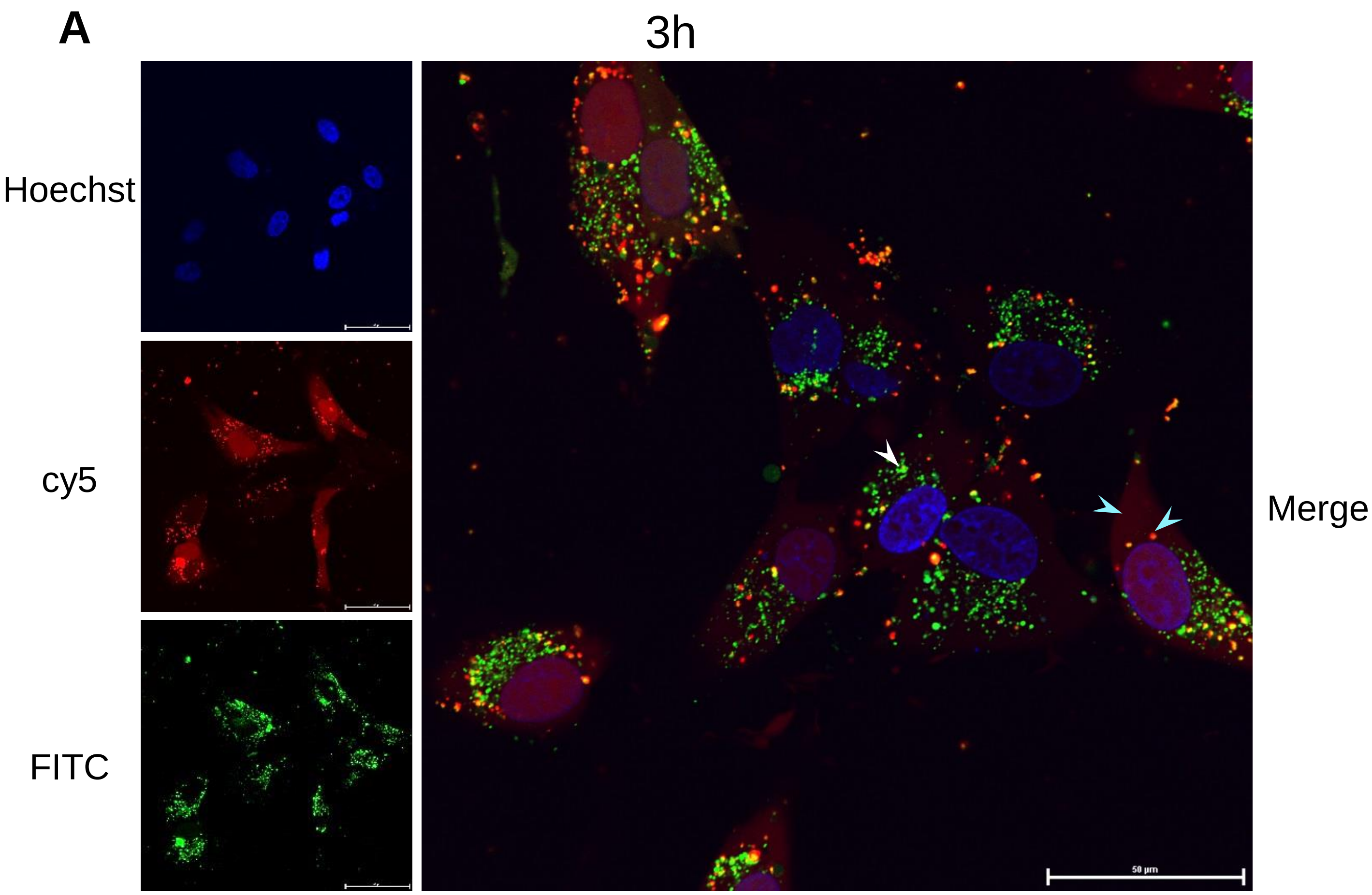
A
3h
Hoechst
cy5
FITC
Merge
50 µm

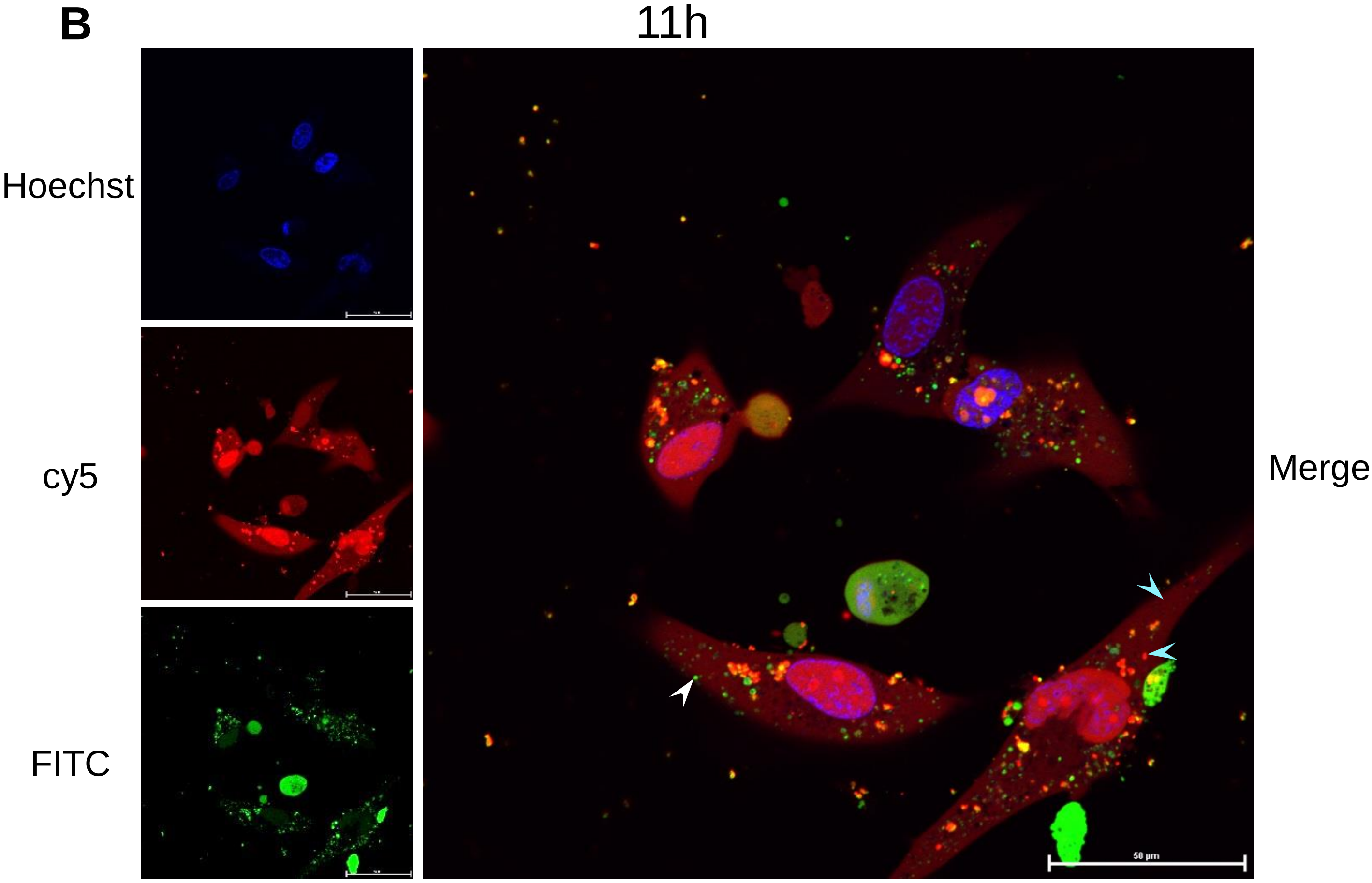

Supplementary Figure 3. Imaging of U87MG cells transfected with cy5 RBFOXi and endosomal labeling with dextran.

Representative images of U87MG cells transfected with 2.5μM cy5 RBFOXi and 0.3mg/ml 488 alexa fluor marked dextran (**A**) 3h (experiment 1) and (**B**) 11h (experiment 2) after transfection and dextran treatment. Images were taken by Nikon SMZ18 stereomicroscope using NIS–Elements Br software. Hoechst (blue), cy5 RBFOXi (red), dextran (green). White line indicates 50μm.

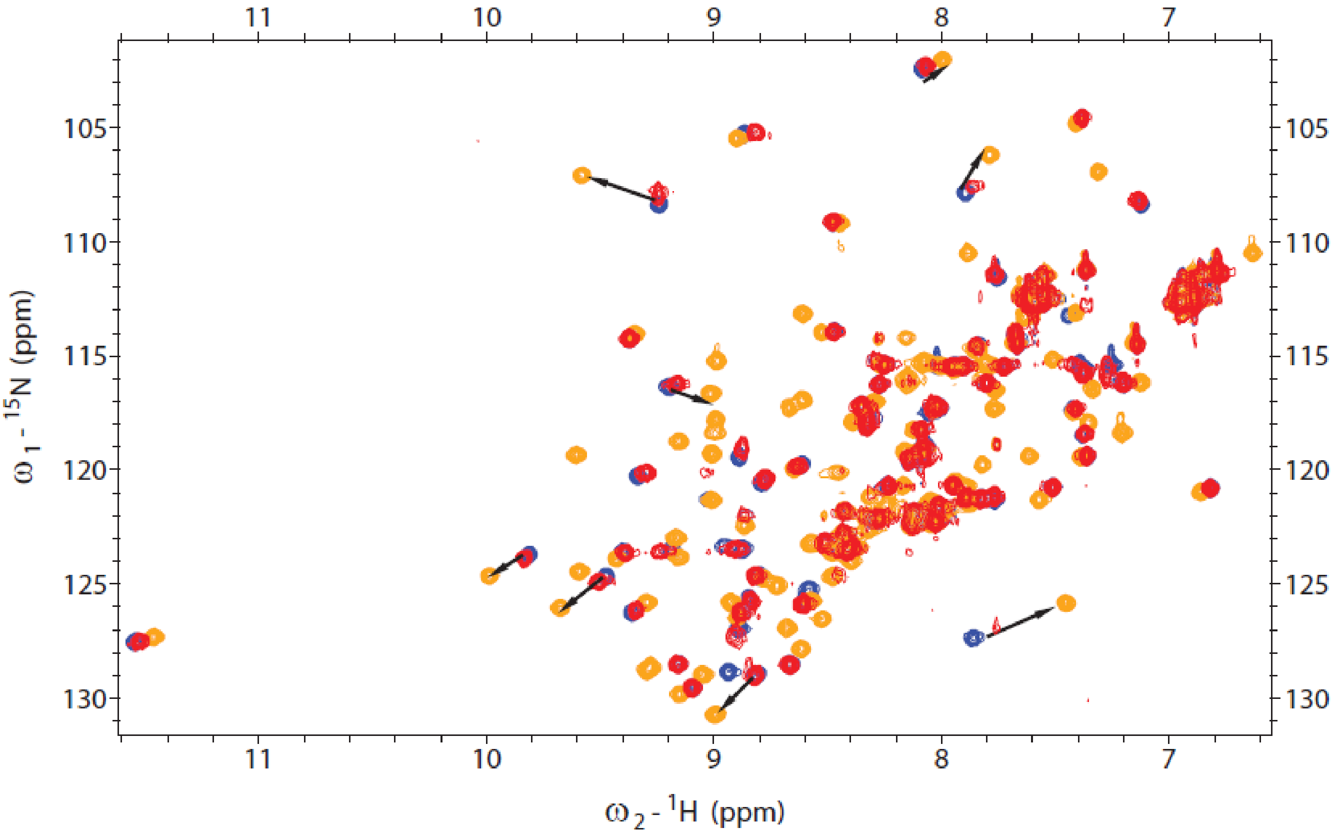

Supplementary Figure 4. NMR titration of RBFOX1 RRM with RBFOXi and UCAGAGGA oligonucleotides.

Overlay of ^{1}H-^{15}N HSQC spectra recorded with RBFOX1 RRM in the free form (in blue), bound to (UGCAUGU) RBFOXi (in orange) and UCAGAGGA (in red) RNAs.

Denichenko et al. Supplementary Figure 4.

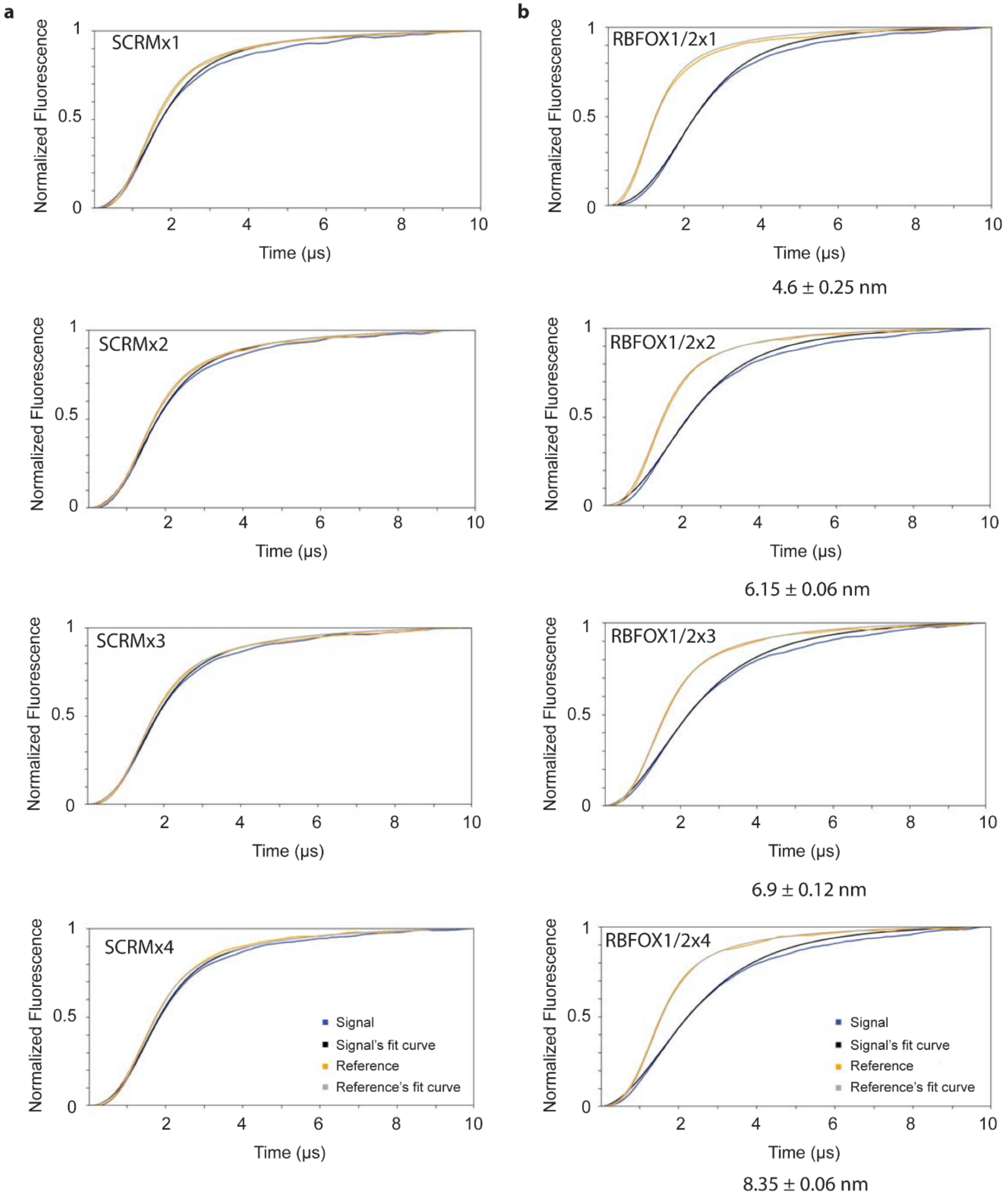

Supplementary Figure 5. Sizing experiments performed with RBFOX1 RRM and oligonucleotides containing one to four consecutive RBFOX1/2 binding sites using switchSENSE

(**A**) Sizing experiments performed using switchSENSE with SCRM×1 - ×4 DNA/RNA and 150nM of RBFOX1 RRM in T40 buffer at 37°C. (**B**) Sizing experiments performed using switchSENSE with RBFOXi×1 - ×4 DNA/RNA and 150nM of RBFOX1 RRM in T40 buffer at 37°C.

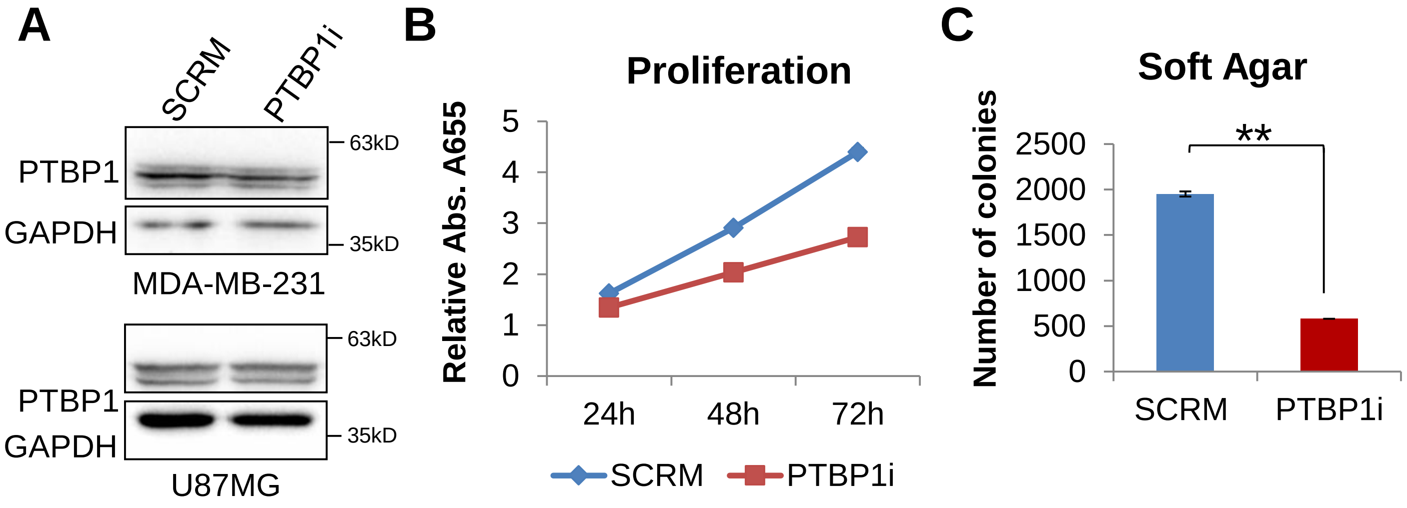

Supplementary Figure 6. Decoy oligonucleotides inhibit biological functions of PTBP1 in a glioblastoma cell line.

(**A**) Western blot of PTBP1 in U87MG and MDA-MB-231 cells transfected with either 5µM biSCRM or biPTBP1i. (**B**) Proliferation assay of U87MG cells transfected with either 5µM biSCRM or biPTBP1i. 4 hours after transfection 4000 cells/well were seeded in six replicates. Cell density was determined every 24 hours (until 72h) by absorbance of methylene blue staining at 655nm. ** p-value < 2.44E-08 for all time points. (**C**) Soft agar colony growth assay on U87MG cells transfected as in (B). Graphs represent quantification of 10 fields counted in duplicate (total of 20 fields). ** p-value < 2.6E-20.

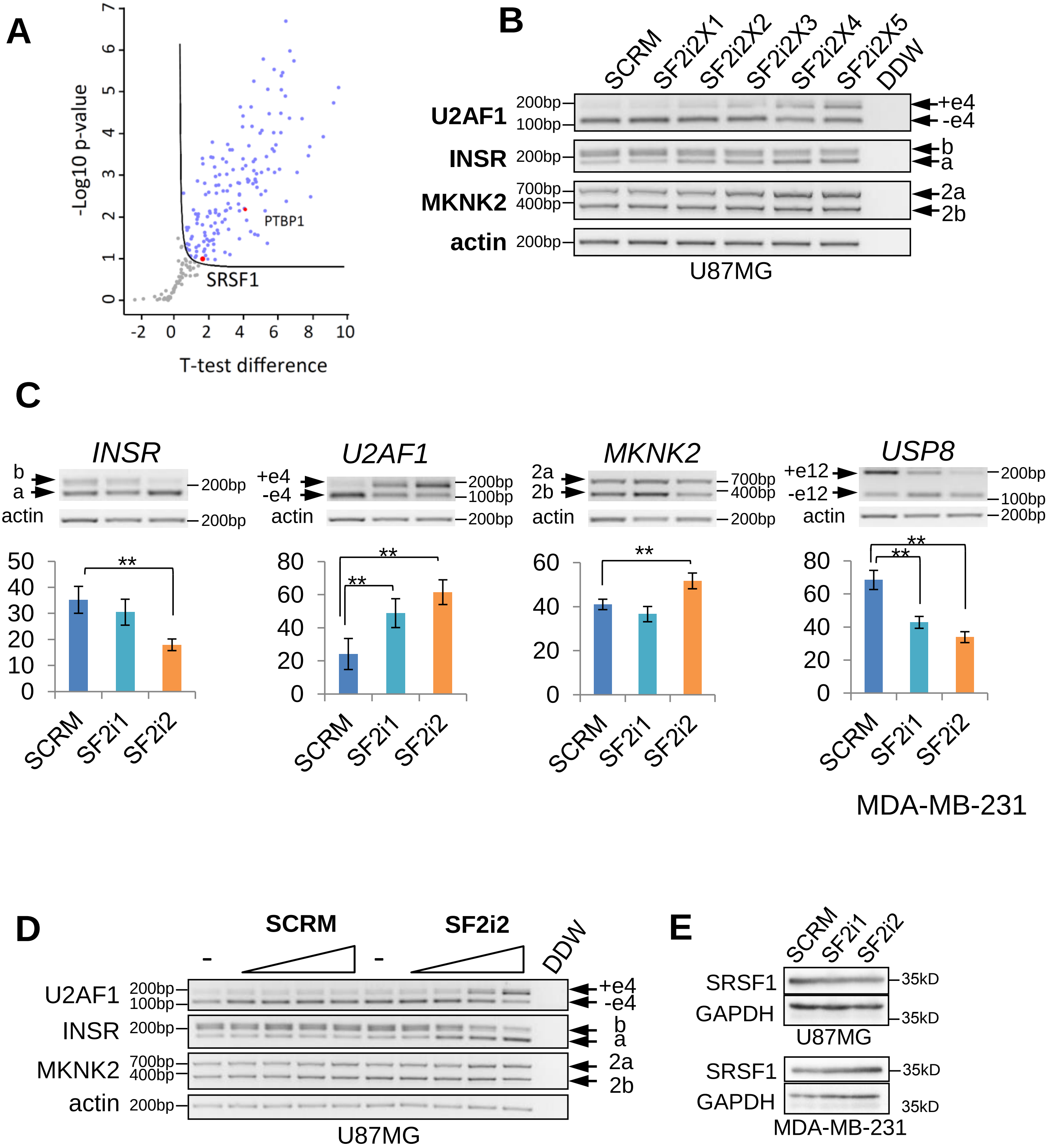

A
-Log10 p-value
T-test difference
PTBP1
SRSF1
B
SCRM
SF2i2X1
SF2i2X2
SF2i2X3
SF2i2X4
SF2i2X5
DDW
U2AF1
INSR
MKNK2
actin
200bp
100bp
700bp
400bp
+e4
-e4
b
a
2a
2b
U87MG
C
INSR
U2AF1
MKNK2
USP8
+e12
-e12
% Inclusion
SCRM
SF2i1
SF2i2
MDA-MB-231
D
SCRM
SF2i2
DDW
U2AF1
INSR
MKNK2
actin
U87MG
E
SCRM
SF2i1
SF2i2
SRSF1
GAPDH
35kD
U87MG
MDA-MB-231

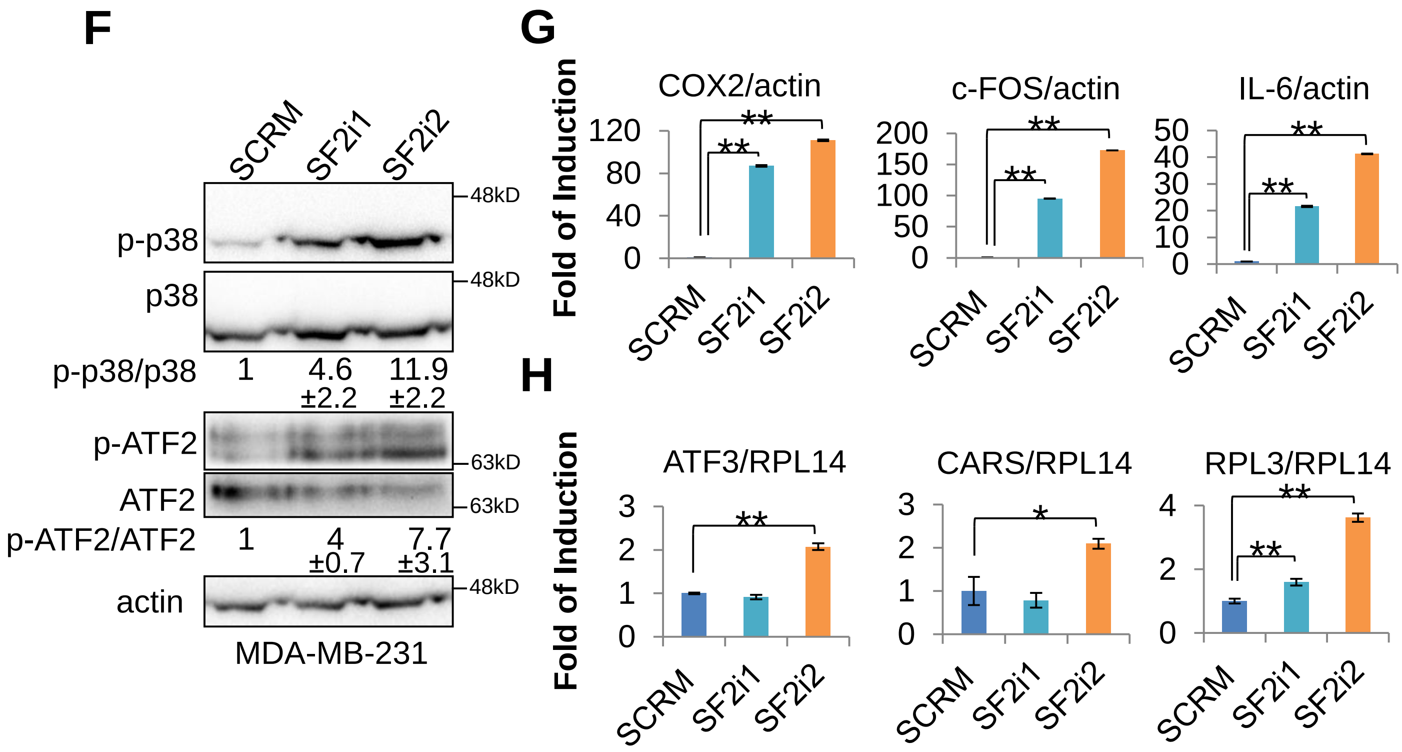

Supplementary Figure 7. SRSF1 decoy oligonucleotides affect splicing of known targets, activate the p38 MAPK pathway and inhibit NMD.

(**A**) Volcano plot showing statistically significant proteins pulled down with biotin conjugated SF2i2, compared to SCRM control, using nuclear extracts from SRSF1 overexpressing HEK293 cells. (**B**) RT-PCR of known splicing targets of SRSF1 in U87MG cells transfected with oligonucleotides containing different number of repeats of the SF2i2 motif. (**C**) RT-PCR and quantification of known splicing targets of SRSF1 in MDA-MB-231 cells transfected with either SCRM or SRSF1 decoy oligonucleotides (SF2i1, SF2i2) (n=6, n=4 for MKNK2 and USP8). Gel image of representative experiment is shown above each column. **p-value < 0.004. (**D**) RT-PCR of known splicing targets of SRSF1 in U87MG cells transfected with increasing concentrations (0.5µM, 1µM, 2.5µM and 5µM) of SCRM or SF2i2. (**E**) Western blot of SRSF1 from U87MG and MDA-MB-231 cells transfected with 2.5µM of either SCRM, SF2i1 or SF2i2. (**F**) Western blot analysis of lysates from MDA-MB-231 cells transfected with 2.5µM of either SCRM, SF2i1 or SF2i2 using the indicated antibodies (n=3). (**G**) RT-qPCR of p38 pathway target genes in cells described in (C). Values are normalized to actin and SCRM value is arbitrarily set to 1. **p-value ≤ 0.001.(**H**) RT-qPCR of NMD targets in cells described in (C). Values are normalized to RPL14 and SCRM value is arbitrarily set to 1. * p-value = 0.02, ** p-value ≤ 0.003.

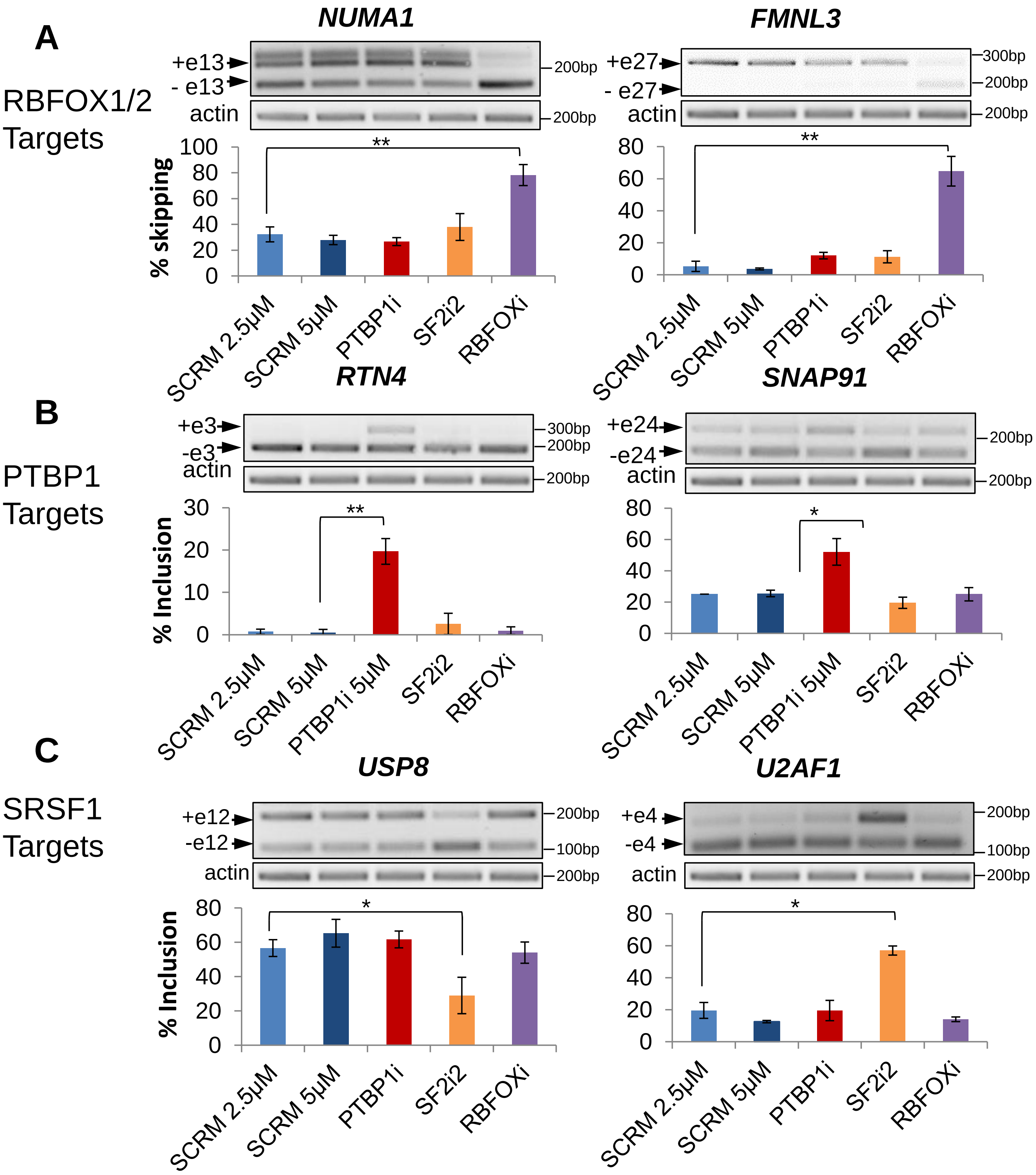

Supplementary Figure 8. Specificity of splicing effects by decoy oligonucleotides.
RT-PCR of known splicing targets of SRSF1 in MDA-MB-231 cells transfected with 2.5µM of SCRM, SF2i2 and RBFOXi and 5µM of SCRM and PTBP1i (n=3). (**A**) RBFOX1/2 targets. (**B**) PTBP1 targets. (**C**) SRSF1 targets. * p-value < 0.03, ** p-value < 0.005.

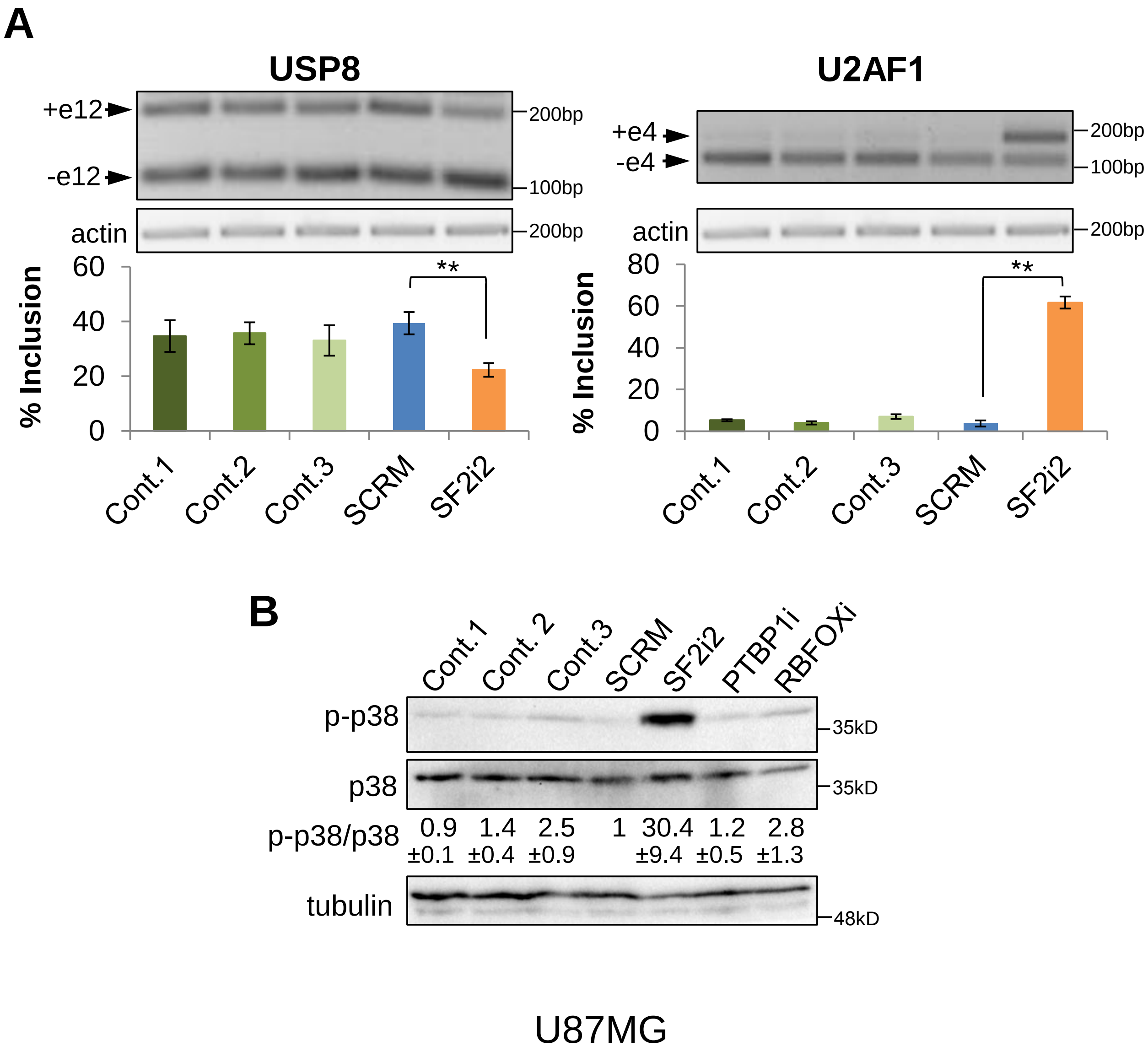

Supplementary Figure 9. Specificity of SRSF1 decoy oligonucleotides in splicing and p38-MAPK pathway activation

U87MG cells were transfected with 2.5μM oligonucleotides and harvested for (A) RNA or (B) protein 48 hours after transfection (n=3). (**A**) RT-PCR of cells transfected with the indicated oligonucleotides. p-value≤0.003. (**B**) Western blot analysis of lysates from cells transfected with the indicated oligonucleotides.

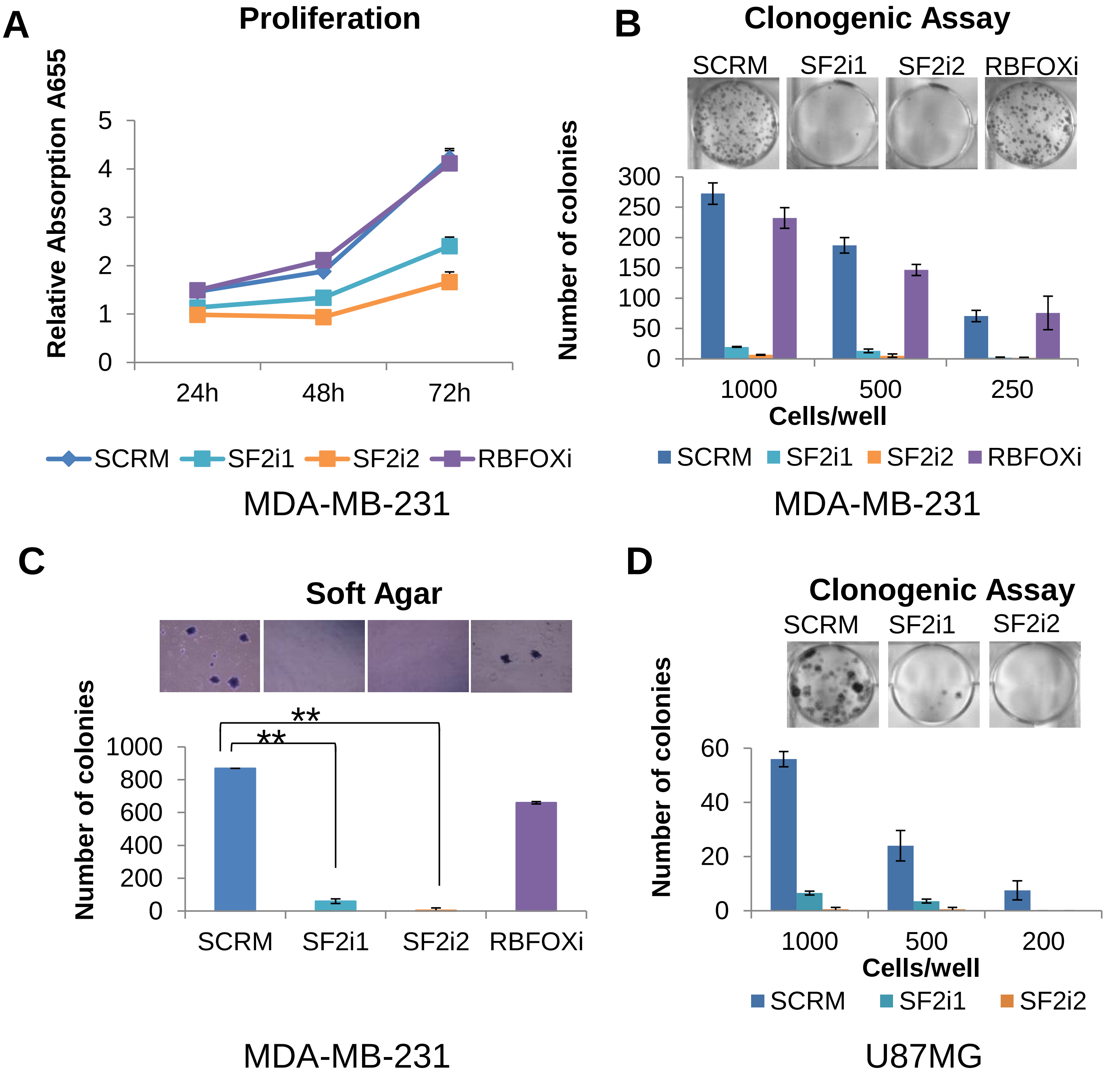

Supplementary Figure 10. SRSF1 decoy oligonucleotides inhibit oncogenic properties of breast cancer and glioblastoma cells while RBFOX1/2 decoy does not.
(**A**) Proliferation assay of MDA-MB-231 cells transfected with 2.5μM of indicated decoy oligonucleotides. 24 hours after transfection cells were seeded 4000 cells/well, six replicates. Cell density was determined every 24 hours (until 72h) by absorbance of methylene blue staining at 655nm. ** p-value ≤ 0.000002 for SF2i1 and SF2i2 for all time points. (**B**) Quantification of clonogenic assay of transfected cells described in (A). Cells were seeded at three different densities. Representative pictures of cells seeded at 1000 cells/well. (**C**) Soft agar colony growth assay of transfected cells described in (A). Graphs represent quantification of 10 fields counted in duplicate (total of 20 fields). **p-value < 1.02E-22. (**D**) Quantification of clonogenic assay of U87MG cells transfected with 2.5μM of SRSF1 decoy oligonucleotide or SCRM. Cells were seeded at three different densities.

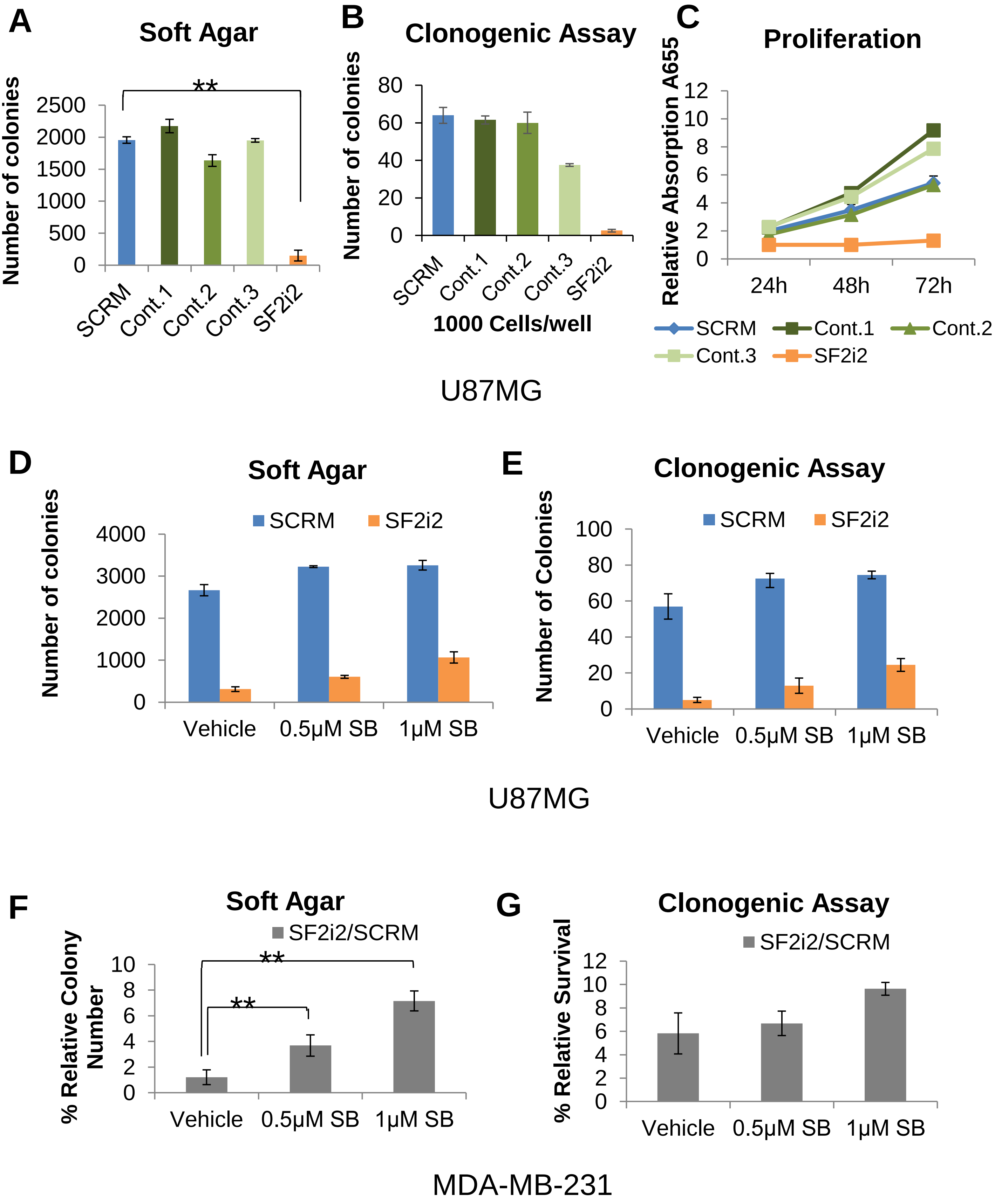
A
Soft Agar
Number of colonies
2500
2000
1500
1000
500
0
**
SCRM
Cont.1
Cont.2
Cont.3
SF2i2
B
Clonogenic Assay
Number of colonies
80
60
40
20
0
SCRM
Cont.1
Cont.2
Cont.3
SF2i2
1000 Cells/well
C
Proliferation
Relative Absorption A655
12
10
8
6
4
2
0
24h
48h
72h
SCRM
Cont.1
Cont.2
Cont.3
SF2i2
U87MG
D
Soft Agar
SCRM
SF2i2
Number of colonies
4000
3000
2000
1000
0
Vehicle
0.5μM SB
1μM SB
E
Clonogenic Assay
SCRM
SF2i2
Number of Colonies
100
80
60
40
20
0
Vehicle
0.5μM SB
1μM SB
U87MG
F
Soft Agar
SF2i2/SCRM
% Relative Colony Number
10
8
6
4
2
0
**
**
Vehicle
0.5μM SB
1μM SB
G
Clonogenic Assay
SF2i2/SCRM
% Relative Survival
12
10
8
6
4
2
0
Vehicle
0.5μM SB
1μM SB
MDA-MB-231

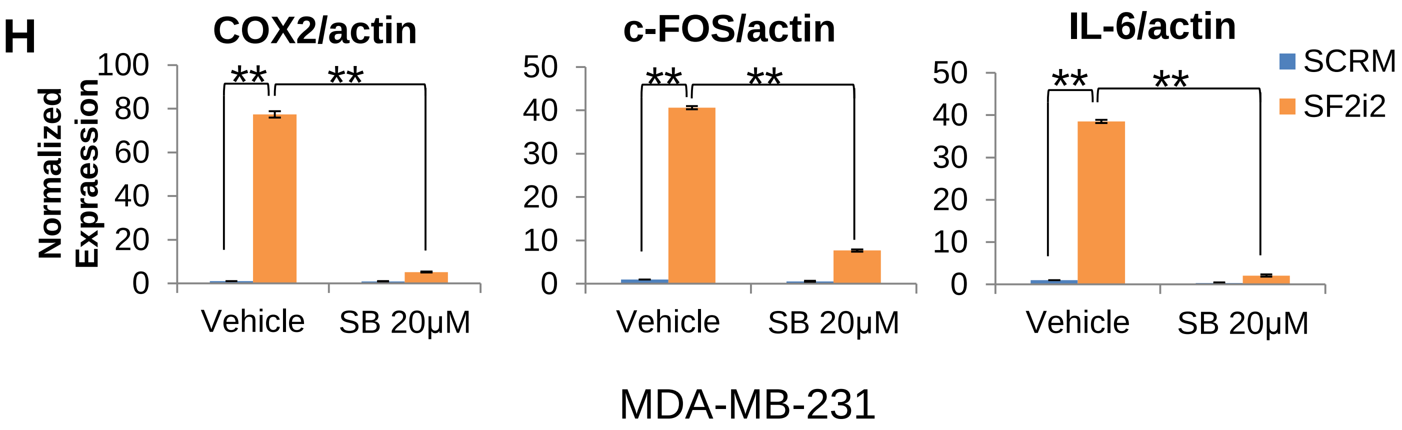

Supplementary Figure 11. SRSF1 decoy oligonucleotides effect on oncogenic properties is partially abrogated by SB203580 treatment.

(A-C) U87MG cells transfected with 2.5μM of indicated oligonucleotides. Cont. 1, Cont. 2, Cont. 3 are antisense oligonucleotides against exon 78 of the dystrophin (DMD) pre-mRNA. (**A**) For soft agar assay, cells were seeded 24h after transfection. Graphs represent quantification of 10 fields counted in duplicate (total of 20 fields). (**B**) For clonogenic assay, 1000 cells were seeded 24h after transfection. (**C**) For proliferation assay, 4 hours after transfection 4000 cells/well were seeded in six replicates. ** p-value < 5.43E-08 for all time points. (**D**) Soft agar colony growth assay of U87MG cells transfected with 2.5μM of either SCRM or SF2i2 with or without SB203580 treatment. Graphs represent quantification of 10 fields counted in duplicate (total of 20 fields). (**E**) Quantification of clonogenic assay of cells described in (D) (1000 cells seeded/well). (**F**) Soft agar colony growth assay of MDA-MB-231 cells transfected with 2.5μM of either SCRM or SF2i2 with or without SB203580 treatment. Graphs represent rescue (relative to SCRM) after treatment with SB203580. (**G**) Clonogenic assay of MDA-MB-231 cells transfected with 2.5μM of either SCRM or SF2i2 with or without SB203580 treatment. Graphs represent rescue (relative to SCRM) after treatment with SB203580. ** p-value < 0.005. (**H**) RT- qPCR of p38 pathway target genes in cells described in (D), normalized to actin and SCRM with or without SB treatment. ** p-value < 0.003.

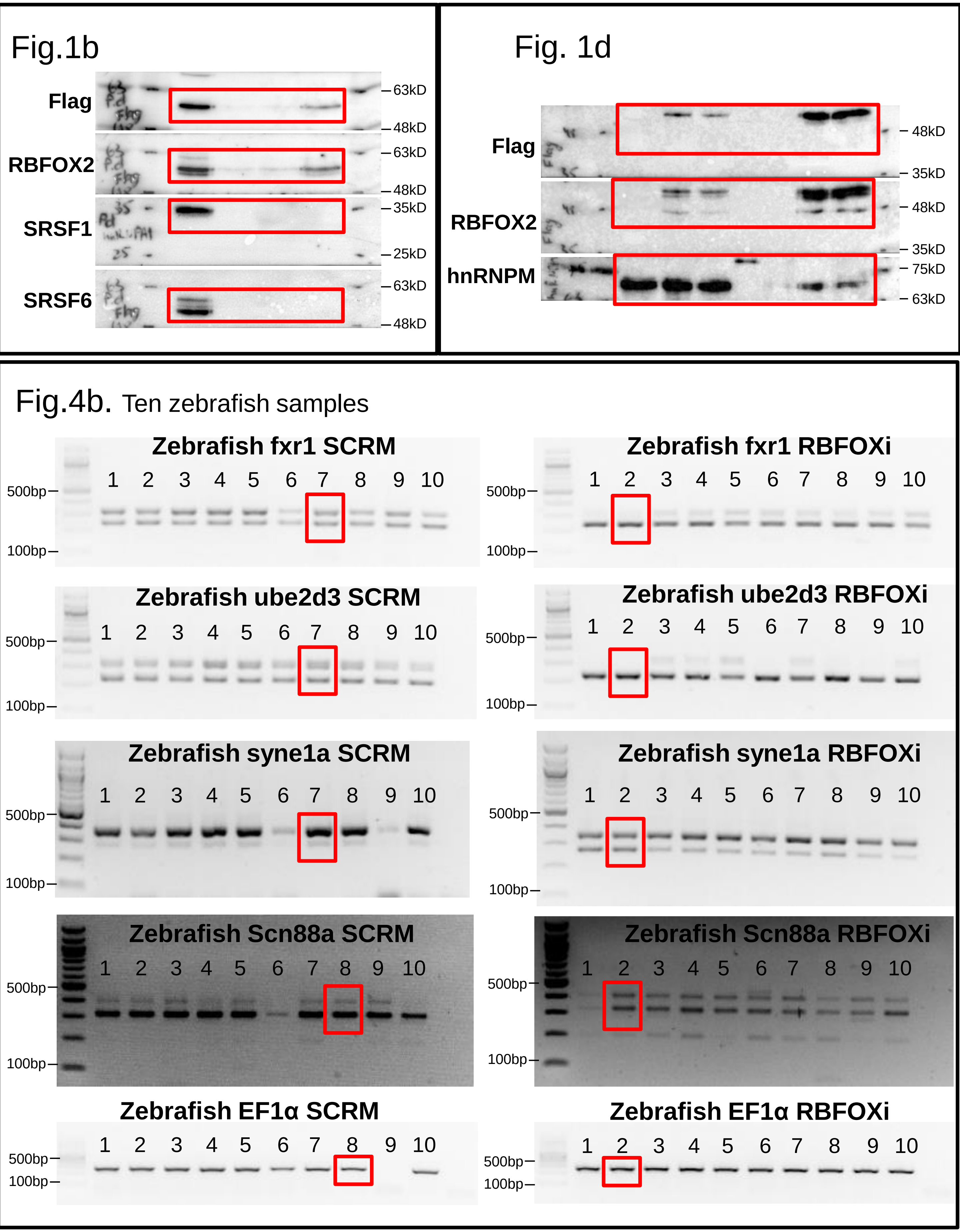
Fig.1b
Flag
RBFOX2
SRSF1
SRSF6
63kD
48kD
63kD
48kD
35kD
25kD
63kD
48kD
Fig. 1d
Flag
RBFOX2
hnRNPM
48kD
35kD
48kD
35kD
75kD
63kD
Fig.4b. Ten zebrafish samples
Zebrafish fxr1 SCRM
Zebrafish fxr1 RBFOXi
Zebrafish ube2d3 SCRM
Zebrafish ube2d3 RBFOXi
Zebrafish syne1a SCRM
Zebrafish syne1a RBFOXi
Zebrafish Scn88a SCRM
Zebrafish Scn88a RBFOXi
Zebrafish EF1α SCRM
Zebrafish EF1α RBFOXi
1 2 3 4 5 6 7 8 9 10
500bp
100bp

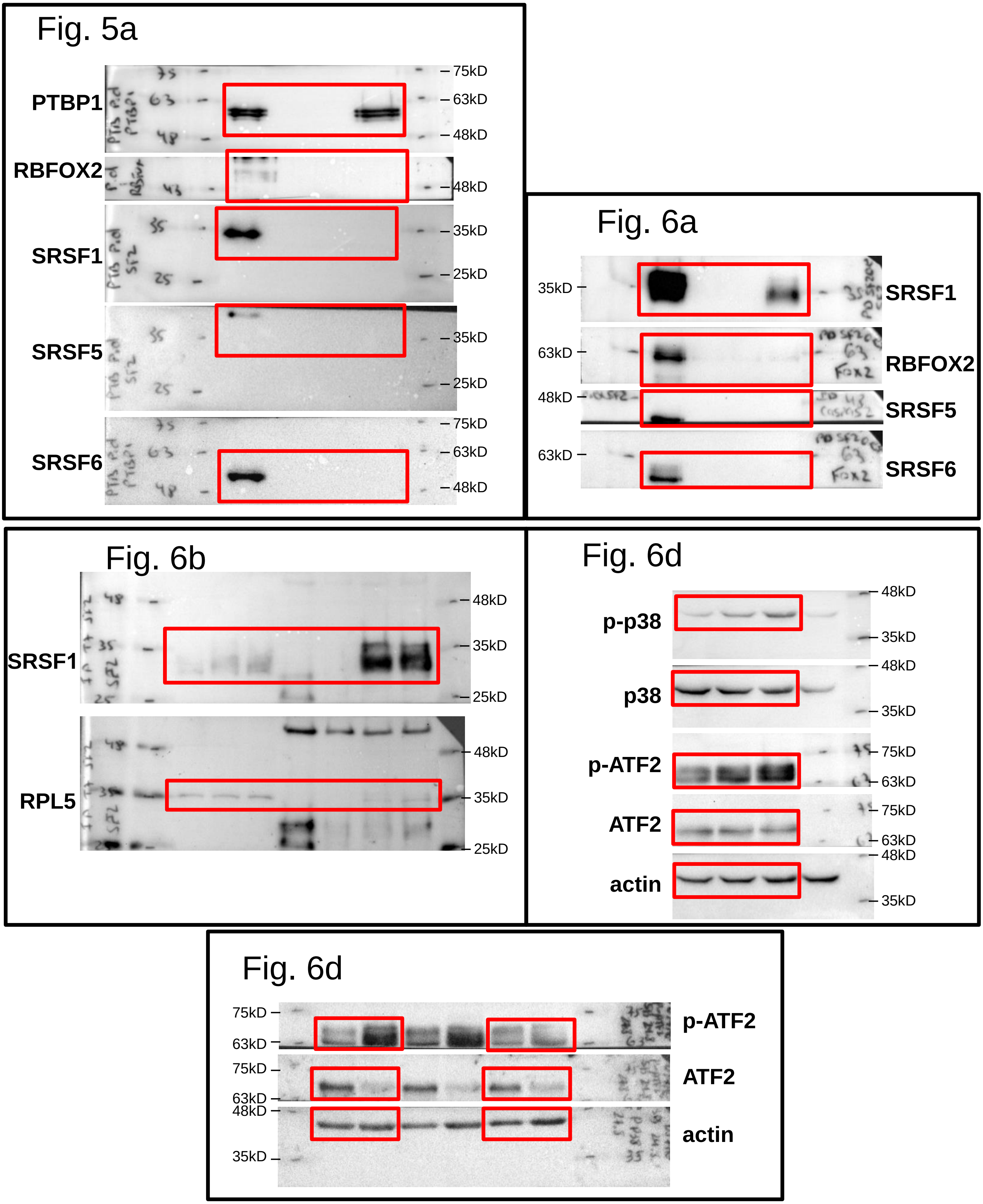

Supplementary Figure 12. Uncropped western blots and PCRs images.
Cropped regions shown in indicated main figures are marked with red boxes.

Supplementary Table 1. Decoy oligonucleotide sequences and RT-PCR Primers

SCRM	5’ (mGmCmAmAmUmCmC)n (n=3)
SF2i1	5’ (mCmAmCmAmGmGmA)n (n=3)
SF2i2	5’ (mCmGmCmAmGmGmA)n (n=3)
RBFOXi	5’ (mUmGmCmAmUmG)n (n=4)
PTBP1i	5’ (mCmUmCmUmCmU)n (n=4)
Cont.1	5’(mAmUmCmUmCmAmCmUmAmAmCmCmUmCmUmCmUmC)
Cont.2	5’(mUmUmCmCmAmGmGmGmGmUmAmUmUmUmCmUmUmC)
Cont.3	5’(mUmGmGmCmUmUmUmCmCmAmGmGmGmGmUmAmUmU)
switchSENSE SCRM	5’ (mGmCmAmAmUmCmC)n TTTTATCA<br>GCGTTCGATGCTTCCGACTAATCAGCC<br>ATATCAGCTTACGACTA (n= 1-4)
switchSENSE<br>RBFOXi	5’ (mUmGmCmAmUmG)n TTTTATCA<br>GCGTTCGATGCTTCCGACTAATCAGCC<br>ATATCAGCTTACGACTA (n= 1-4)
Human INSR	For- AGATCCTGAAGGAGCTGGAGGA<br>Rev- GGTCGAGGAAGTGTTGGG
Human MKNK2	For- GCTGCGACCTGTGGAGCCTGGG<br>Rev- GATGGGAGGGTCAGGCGTGGTC<br>Rev- GAGGAGGAAGTGACTGTCCCAC
Human U2AF1	For- CGTAATCCCCAAAACAGTGC<br>Rev- TCATCATAGTGTTCCTGCATCTC
Human ATF3	For- GCCATTGGAGAGCTGTCTTC<br>Rev- GGGCCATCTGGAACATAAGA
Human CARS	For- AAATTAAATGAGACCACGGA<br>Rev- TGACATCACAGCCAAGTGTA
Human RPL3	For- GGCATTGTGGGCTACGTG<br>Rev- CTTCAGGAGCAGAGCAGA
Human c-FOS	For- CTGTCAACGCGCAGGACTT<br>Rev- GGGGCTCTGGTCTGCGAT
Human IL-6	For- CAAATTCGGTACATCCTCGAC<br>Rev- GAAGGTTCAGGTTGTTTTCTG
Human COX2	For- CCGAGGTGTATGTATGAGTGT<br>Rev- CTGTGTTTGGAGTGGGTTTC
Human ABI1	For - TTCCCAGTATGGCACAATGA<br>Rev - CCAAGCAGGATCCCCATCTGC
Human FMNL3	For - GAACACCGGCCTGTTTATGAG<br>Rev- AAGTGCTTCTGCCTCCGAGAG
Human FAM126A	For- TCAATAAGGGGTCATAGGTGG

	Rev- AGGCTTAGGTAGGGATTGGC
Human NUMA1	For- GGAGCTGGAGGTGATGACTGC<br>Rev- CTTCAGCTTCTGCTGCTGCAC
Human β-actin	For- CGTGGACATCCGCAAAG<br>Rev- GGAAGGTGGACAGCGAG
Human SNAP91	For- AGCAGCCGGTCATGTTTGCAC<br>Rev-ACTCAGCATCAATCTTATTTGAAGTCTC
Human PKM2	For- GCTGGAGAGCATGATCAAGA<br>Rev- ACTTGAGGCTCGCACAAGTT<br>Rev- AGGACGATTATGGCCCCACT
Human RTN4	For- CTGCATCTGAGCCTGTGATA<br>Rev- GATTCTGCTTTTGGAGAGACAC
Human PTBP2	For- AGCTGCTGCTGGCCGAGTG<br>Rev- GATTGGTTTCCATCAGCCATCTG
Zebrafish fxr1	For- CACCGATGAGGACACCACAGTC<br>Rev- GGTTCGTCTGCGTTATTGGGGC
Zebrafish ube2d3	For- GTCCATCTGCTCACTCTTATGTGAC<br>Rev- CCTCCATAAGAGACAGATGGGGAAA
Zebrafish syne1a	For- GGTCAGACAGAAAGATGAGGATGAG<br>Rev- TTATCAGGGTTGCTCAGGCCTGACA
Zebrafish scn88a	For- CAAGTACTTCACCAATGCTTGGTGC<br>Rev- CGTTGTAGCAGTAGTAGTAC
Zebrafish Ef1α	For- AAGACAACCCCAAGGCTCTCA<br>Rev- CCTTTGGAACGGTGTGATTGA
