# Supplementary material for: Specific inhibition of splicing factor activity by decoy RNA oligonucleotides
Source: Nat Commun. 2019 Apr 8;10:1590. doi: 10.1038/s41467-019-09523-0 (PMC6453957; doi:10.1038/s41467-019-09523-0)
Supplement: Supplementary file 3 — Description of Additional Supplementary Files [file 41467_2019_9523_MOESM3_ESM.pdf]

**Description of Additional Supplementary Files**

File Name: Supplementary Movie 1
Description: cy5 RBFOXi+Dextran; 19.5 hours post transfection, with a gap between 12.5 to 15 hours due to lens focus escape

File Name: Supplementary Movie 2
Description: cy5 RBFOXi+Dextran_2; 21.5 hours post transfection, with a gap between 10 to 12.5 hours due to lens focus escape

File Name: Supplementary Movie 3
Description: cy5 RBFOXi+Dextran_3; 18 hours post transfection

File Name: Supplementary Data 1
Description: Quantification of Cy5-labeled FBFOXi decoy in cytoplasm and nucleus 24 hours post transfection

File Name: Supplementary Data 2
Description: Proteomic analysis of proteins bound by the biotin-RBFOXi decoy oligonucleotide

File Name: Supplementary Data 3
Description: RNA-seq analysis following transfection with FBFOXi decoy oligonucleotide and comparison to Damianov et al.

File Name: Supplementary Data 4
Description: Proteomic analysis of proteins bound by the biotin-PTBP1i decoy oligonucleotide

File Name: Supplementary Data 5
Description: Proteomic analysis of proteins bound by the biotin-SF2i2 decoy oligonucleotide
