# Supplementary material for: Specific inhibition of splicing factor activity by decoy RNA oligonucleotides
Source: Nat Commun. 2019 Apr 8;10:1590. doi: 10.1038/s41467-019-09523-0 (PMC6453957; doi:10.1038/s41467-019-09523-0)
Supplement: Supplementary file 12 — Reporting Summary [file 41467_2019_9523_MOESM12_ESM.pdf]

nature research

Corresponding author(s): Rotem Karni

Last updated by author(s): Feb 6, 2019

Reporting Summary

Nature Research wishes to improve the reproducibility of the work that we publish. This form provides structure for consistency and transparency in reporting. For further information on Nature Research policies, see Authors & Referees and the Editorial Policy Checklist.

Statistics

For all statistical analyses, confirm that the following items are present in the figure legend, table legend, main text, or Methods section.

n/a	Confirmed	
☐	☒	The exact sample size (*n*) for each experimental group/condition, given as a discrete number and unit of measurement
☒	☐	A statement on whether measurements were taken from distinct samples or whether the same sample was measured repeatedly
☐	☒	The statistical test(s) used AND whether they are one- or two-sided<br>*Only common tests should be described solely by name; describe more complex techniques in the Methods section.*
☐	☒	A description of all covariates tested
☐	☒	A description of any assumptions or corrections, such as tests of normality and adjustment for multiple comparisons
☐	☒	A full description of the statistical parameters including central tendency (e.g. means) or other basic estimates (e.g. regression coefficient) AND variation (e.g. standard deviation) or associated estimates of uncertainty (e.g. confidence intervals)
☐	☒	For null hypothesis testing, the test statistic (e.g. *F*, *t*, *r*) with confidence intervals, effect sizes, degrees of freedom and *P* value noted<br>*Give P values as exact values whenever suitable.*
☒	☐	For Bayesian analysis, information on the choice of priors and Markov chain Monte Carlo settings
☒	☐	For hierarchical and complex designs, identification of the appropriate level for tests and full reporting of outcomes
☒	☐	Estimates of effect sizes (e.g. Cohen's *d*, Pearson's *r*), indicating how they were calculated

Our web collection on statistics for biologists contains articles on many of the points above.

Software and code

Policy information about availability of computer code

Data collection	*Provide a description of all commercial, open source and custom code used to collect the data in this study, specifying the version used OR state that no software was used.*
Data analysis	*Provide a description of all commercial, open source and custom code used to analyse the data in this study, specifying the version used OR state that no software was used.*

For manuscripts utilizing custom algorithms or software that are central to the research but not yet described in published literature, software must be made available to editors/reviewers. We strongly encourage code deposition in a community repository (e.g. GitHub). See the Nature Research guidelines for submitting code & software for further information.

Data

Policy information about availability of data

All manuscripts must include a data availability statement. This statement should provide the following information, where applicable:

- Accession codes, unique identifiers, or web links for publicly available datasets
- A list of figures that have associated raw data
- A description of any restrictions on data availability

RNA-seq data have been deposited to the GEO repository as GEO submission GSE126503. The mass spectrometry proteomics data have been deposited to the ProteomeXchange Consortium via the PRIDE [1] partner repository with the dataset identifier PXD012564. The authors declare that all the other data supporting the findings of this study are available within the article and its supplementary information files and from the corresponding author upon reasonable request. The source data underlying Figs 1b, 1d, 4b, 5a, 6a, 6b and 6d, are provided in Supplementary Figure 12.

Field-specific reporting

Please select the one below that is the best fit for your research. If you are not sure, read the appropriate sections before making your selection.

☒ Life sciences ☐ Behavioural & social sciences ☐ Ecological, evolutionary & environmental sciences

For a reference copy of the document with all sections, see nature.com/documents/nr-reporting-summary-flat.pdf

Life sciences study design

All studies must disclose on these points even when the disclosure is negative.

Sample size	Each experiment was performed in duplicates at least, and repeated at least three times. Average, standard deviation and p-values are shown for each experiment and calculated by two tailed student T-test.
Data exclusions	No data exclusion
Replication	Each experiment was performed in duplicates at least, and repeated at least three times (for many experiments there are even 6-7 seperated repeats.
Randomization	Not relevant to this study
Blinding	Not relevant to this study

Reporting for specific materials, systems and methods

We require information from authors about some types of materials, experimental systems and methods used in many studies. Here, indicate whether each material, system or method listed is relevant to your study. If you are not sure if a list item applies to your research, read the appropriate section before selecting a response.

Materials & experimental systems

n/a	Involved in the study	
☐	☒	Antibodies
☐	☒	Eukaryotic cell lines
☒	☐	Palaeontology
☐	☒	Animals and other organisms
☒	☐	Human research participants
☒	☐	Clinical data

Methods

n/a	Involved in the study	
☒	☐	ChIP-seq
☒	☐	Flow cytometry
☒	☐	MRI-based neuroimaging

Antibodies

Antibodies used	Primary antibodies: RBFOX2 (1:4000, Sigma), SRSF1 (1:200, mAb AK96 culture supernatant 57), SRSF5 (1:3000, Sigma), SRSF6 (1:500, mAb 8-1-28 culture supernatant), OctA (Flag tag) (1:500, Santa Cruz), hnRNPM (1:500, Novusbio), PTBP1 (1:10,000, Abcam), T7 (1:5000 Novagen), phospho-p38 (Thr180/Tyr182) (1:1000 cell signaling), p38 (1:1000 Santa Cruz), phospho- ATF2 (Thr71) (1:1000 Cell Signaling), ATF2 (1:1000 abcam), β tubulin I+II (1:1000, Sigma), β-actin (1:200, Santa Cruz), GAPDH (1:1000, Santa Cruz), Secondary antibodies: HRP-conjugated goat anti-mouse, goat anti-rabbit or donkey anti-goat IgG (H+L) (1:10,000, Jackson Laboratories).
Validation	All the antibodies used were either commercially available and specification can be found in the manufacturer data sheet. SRSF1 (1:200, mAb AK96 culture supernatant 57) and SRSF6 (1:500, mAb 8-1-28 culture supernatant) were described in previous publications (Karni et al. 2007, Nature Struc Mol Biol. 14:185-93); (Cohen-Eliav M. et al. J Pathol. 2013 229:630-9).

Eukaryotic cell lines

Policy information about cell lines

Cell line source(s)	All cell lines source was ATCC and they were validated using genomic DNA analysis by Bio-Synthesis, Inc.
Authentication	All cells were validated using genomic DNA analysis by Bio-Synthesis, Inc.
Mycoplasma contamination	All cells were routinely (every two weeks) examined for mycoplasma by a PCR-based Mycoplasma test kit (Biological industries, Israel)

Commonly misidentified lines
(See ICLAC register)

Some experiments (which were repeated by another cell lines in all cases) were performed on MDA-MB-435s cells - These experiments are indicated in the text and figure legends.

Animals and other organisms

Policy information about studies involving animals; ARRIVE guidelines recommended for reporting animal research

Laboratory animals	Mice experiments were done on female NOD-SCID mice.
Wild animals	No wild animals
Field-collected samples	No Field-collected samples
Ethics oversight	All animal experiments were performed in accordance with the guidelines of the Hebrew University committee for the use of animals for research. All experiments complied with ethical regulations for animal testing and research, Hebrew University (IACUC) Ethics approval no' MD-15-14634-5. HU is AAALAC approved.

Note that full information on the approval of the study protocol must also be provided in the manuscript.
